# The Widely Used Antimicrobial Triclosan Induces High Levels of Antibiotic Tolerance *In Vitro* and Reduces Antibiotic Efficacy up to 100-Fold *In Vivo*

**DOI:** 10.1128/AAC.02312-18

**Published:** 2019-04-25

**Authors:** Corey Westfall, Ana Lidia Flores-Mireles, John Isaac Robinson, Aaron J. L. Lynch, Scott Hultgren, Jeffrey P. Henderson, Petra Anne Levin

**Affiliations:** a Department of Biology, Washington University in St. Louis, St. Louis, Missouri, USA; b Department of Molecular Microbiology and Microbial Pathogenesis, Washington University School of Medicine, St. Louis, Missouri, USA; c Division of Infectious Diseases, John T. Milliken Department of Internal Medicine, Washington University School of Medicine, St. Louis, Missouri, USA; d Department of Biological Sciences, University of Notre Dame, Notre Dame, Indiana, USA

**Keywords:** antimicrobial agents, antimicrobial safety, genetics, urinary tract infection

## Abstract

The antimicrobial triclosan is used in a wide range of consumer products ranging from toothpaste, cleansers, socks, and baby toys. A bacteriostatic inhibitor of fatty acid synthesis, triclosan is extremely stable and accumulates in the environment. Approximately 75% of adults in the United States have detectable levels of the compound in their urine, with a sizeable fraction of individuals (>10%) having urine concentrations equal to or greater than the minimal inhibitory concentration for Escherichia coli and methicillin-resistant Staphylococcus aureus (MRSA). Previous work has identified connections between defects in fatty acid synthesis and accumulation of the alarmone guanosine tetraphosphate (ppGpp), which has been repeatedly associated with antibiotic tolerance and persistence. Based on these data, we hypothesized that triclosan exposure may inadvertently drive bacteria into a state in which they are able to tolerate normally lethal concentrations of antibiotics. Here we report that clinically relevant concentrations of triclosan increased E. coli and MRSA tolerance to bactericidal antibiotics as much as 10,000-fold *in vitro* and reduced antibiotic efficacy up to 100-fold in a mouse urinary tract infection model. Genetic analysis indicated that triclosan-mediated antibiotic tolerance requires ppGpp synthesis but is independent of growth. These data highlight an unexpected and certainly unintended consequence of adding high concentrations of antimicrobials in consumer products, supporting an urgent need to reevaluate the costs and benefits of the prophylactic use of triclosan and other bacteriostatic compounds.

## Abstract (precis)

The antimicrobial triclosan is used in a wide range of consumer products ranging from toothpaste, cleansers, socks, and baby toys. A bacteriostatic inhibitor of fatty acid synthesis, triclosan is extremely stable and accumulates in the environment.

## INTRODUCTION

The prophylactic use of antibiotics in consumer goods ranging from animal feed to personal care products is widely believed to be a major contributor to the epidemic increase in antibiotic-resistant pathogens (1–3). Prominent among these prophylactics are triclosan and triclocarban, polychlorinated aromatic antimicrobials targeting fatty acid synthesis. Triclosan in particular is found in a wide variety of consumer products, including toothpaste, cleansers, socks, and baby toys (1). Although the U.S. Food and Drug Administration effectively banned the use of triclosan in household soap in 2017, as of this writing Canada and Australia, among other countries, have not elected to take similar actions.

An inhibitor of enoyl-acyl carrier protein reductase (4) at low concentrations (∼200 ng/ml for E. coli), triclosan is bacteriostatic, preventing cell growth but having little effect on viability over the short term. At high concentrations (>10 μg/ml), triclosan is bactericidal, most likely killing cells through disruption of plasma membrane integrity (4). Recent work from the Waters lab suggests that at these higher concentrations triclosan can serve as an adjuvant, acting synergistically with tobramycin and other drugs, increasing killing by ∼100-fold in a Pseudomonas aeruginosa biofilm model for cystic fibrosis lung infections (5). Triclosan is typically used as an antimicrobial additive at these higher, bactericidal concentrations.

Because of its widespread use as a prophylactic, the high concentrations at which it is employed, and its inherent stability, triclosan accumulates to high levels in the environment (6, 7). Approximately 75% of adults in the United States have detectable levels of the compound in their urine, and >10% have urine concentrations greater than or equal to the MIC for Escherichia coli (200 ng/ml) and methicillin-resistant Staphylococcus aureus (MRSA; 100 ng/ml) (8, 9).

While the inverse relationship between antibiotic use and antibiotic efficacy is largely attributable to the selection of heritable traits, nonheritable traits such as antibiotic tolerance and persistence are also likely to be involved (10). In contrast to genetically resistant bacteria, which grow in the presence of an antibiotic, tolerant bacteria are able to survive antibiotic challenge for longer periods of time than their more sensitive counterparts (10). Persister cells are the small subset of an otherwise-sensitive population (∼1 in 10^6^) that exhibit levels of tolerance sufficient to protect them from otherwise lethal concentrations of antimicrobial compounds (11). Increases in antibiotic tolerance and persistence are confounding factors in the treatment of chronic P. aeruginosa (12) and S. aureus (13) infections and are thought to contribute to the refractory nature of medically relevant biofilms (14). Reduced growth rate and metabolic activity is associated with increased antibiotic tolerance (10) and is a defining trait of persister cells.

Based on previous work identifying connections between defects in fatty acid synthesis and accumulation of the alarmone guanosine tetraphosphate (ppGpp) (15), as well as reports of links between ppGpp and antibiotic tolerance (16, 17), we hypothesized that triclosan exposure may inadvertently drive bacteria into a metabolically depressed state in which they are able to tolerate normally lethal concentrations of antibiotics (18, 19). In particular, inhibiting fatty acid synthesis stimulates interaction between acyl carrier protein and the hydrolase domain of the bifunctional ppGpp synthase SpoT, resulting in accumulation of the alarmone and the concomitant inhibition of biosynthetic capacity (20).

Here we report that clinically relevant bacteriostatic concentrations of triclosan increased E. coli and methicillin-resistant S. aureus (MRSA) tolerance to bactericidal antibiotics as much as 10,00-fold *in vitro* and reduced antibiotic efficacy ∼100-fold in a mouse urinary tract infection (UTI) model. Triclosan-mediated antibiotic tolerance is dependent on ppGpp synthesis: although triclosan inhibited the growth of both wild-type and ppGpp mutant cells, only the latter were highly susceptible to challenge with bactericidal compounds. In contrast, pretreatment with another bacteriostatic drug, spectinomycin, a translation inhibitor that does not impact ppGpp accumulation (21), induced high levels of antibiotic tolerance in both wild-type and ppGpp mutant cells. Together, these data highlight an unexpected and certainly unintended consequence of employing triclosan as a commercial antimicrobial and support an urgent need to reevaluate the costs and benefits of the addition of triclosan and potentially other bacteriostatic compounds to consumer products.

## RESULTS

### Triclosan pretreatment results in high levels of tolerance to bactericidal antibiotics *in vitro*.

To assess whether physiologically relevant levels of triclosan are sufficient to promote tolerance to bactericidal antibiotics, we examined the relative sensitivity of E. coli (MG1655) and S. aureus (FPR3757 an USA-300 MRSA strain) cultured in MICs of triclosan to a panel of bactericidal antibiotics. The triclosan MICs for E. coli and MRSA were 200 and 100 ng/ml, respectively, under our growth conditions, similar to the triclosan concentration found in the urine from individuals using triclosan-containing products (8, 9). In all cases, triclosan was added 30 min prior to the addition of the specified bactericidal antibiotic, and both antibiotics were maintained in the culture for the remainder of the experiment.

**FIG 1:**
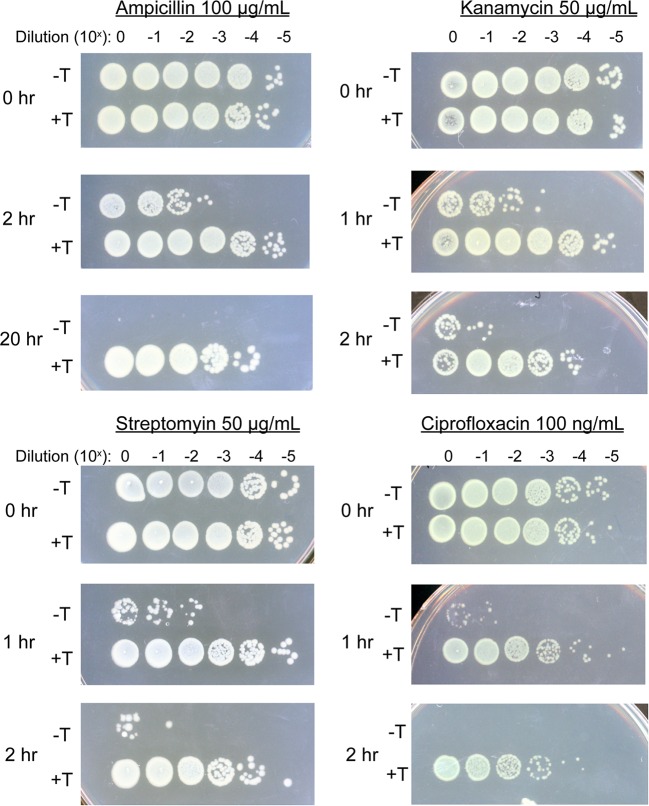
Triclosan induces tolerance to multiple antibiotics. E. coli (MG1655) were cultured to an OD_600_ of 0.2, split, and cultured for an additional 30 min with (+T) or without (–T) 200 ng/ml triclosan. The indicated bactericidal antibiotics were then added, and the cells were cultured for an additional 2 to 20 h prior to dilution plating. Each experiment was replicated three independent times, with only representative data shown.

Triclosan had a dramatic protective effect on E. coli in an endpoint assay, increasing survival by several orders of magnitude in the presence of three bactericidal antibiotics and providing nearly complete protection against a fourth (Fig. 1). E. coli treated with triclosan exhibited a 1,000-fold increase in survival in the presence of 50 μg/ml (∼5× MIC) kanamycin, an inhibitor of peptide bond formation. Triclosan treated cells also showed a 10,000-fold increase in survival in the presence of streptomycin (50 μg/ml; ∼2× MIC), an inhibitor of tRNA-ribosome interaction, and ciprofloxacin (100 ng/ml; ∼3× MIC), a gyrase inhibitor (Fig. 1). Strikingly, triclosan rendered E. coli almost completely refractory to treatment with the cell wall active antibiotic ampicillin (100 μg/ml; ∼10× MIC). Viable cell numbers were essentially identical in triclosan-treated and triclosan-ampicillin-treated cultures at 2 h, and 10% of cells in triclosan-ampicillin cultures were viable at 20 h, suggesting that triclosan increased persister frequency to all tested antibiotics.

**FIG 2:**
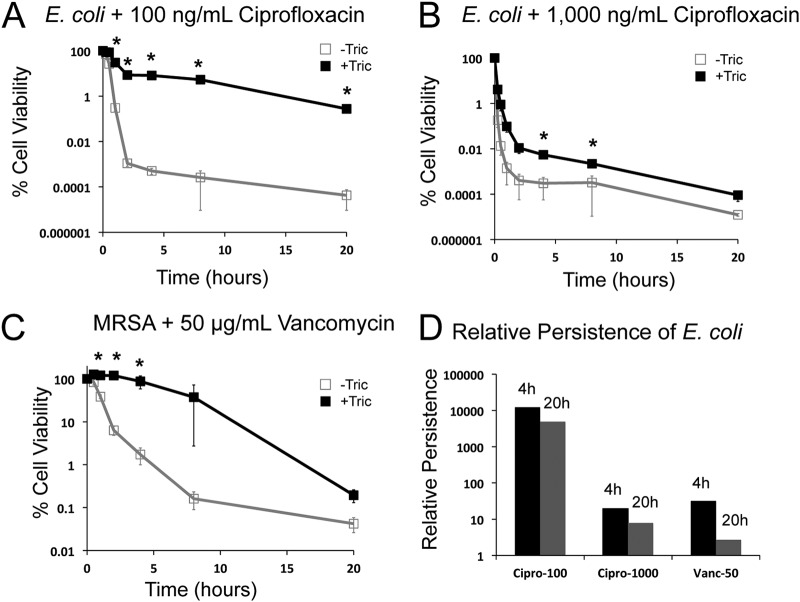
Kinetic analysis of triclosan-induced persistence. E. coli (MG1655) and MRSA (FPR3757) cells were cultured to an OD_600_ of 0.2, split and cultured for an additional 30 min with (black line, closed squares) or without triclosan (gray line, open squares). (A to C) At *t* = 0, 100 ng/ml (A) or 1,000 ng/ml ciprofloxacin (B) was added to the E. coli cultures, and 50 ng/ml vancomycin was added to the MRSA cultures (C). (D) The relative persistence in the presence of triclosan (CFU+T/CFU–T) was calculated from the 4- and 20-h time points. Values are the means of three independent biological replicates, with error bars representing the SEM. Asterisks represent significant differences between triclosan-treated and nontreated samples calculated using a Student two-tailed *t* test (*, *P* < 0.05).

Triclosan also protected MRSA cells from high concentrations of the glycopeptide antibiotic, vancomycin, over the course of a 20-h experiment (Fig. 2C). MRSA treated with 100 ng/ml of triclosan were essentially refractory to 50 ng/ml vancomycin (10× MIC) at 4 h and exhibited a viable cell count ∼200 times that of untreated cells at 8 h. The viable cell count, even at 20 h, was several times higher in the presence of both triclosan and vancomycin than vancomycin alone. This delayed reduction in viable cell count is consistent with induction of a persistent state (10).

### Triclosan increases persister cell frequency.

To further assess the protective effect of triclosan, we performed kinetic kill curves, in which we measured the CFU over a 20-h time frame. If persister cells are present, we expect to observe two slopes, one corresponding to the kill rate of the general population and the second slope corresponding to the slower kill rate of persister cells (10). In this manner, the kill curve is able to separate antibiotic tolerance at the population level from the impact of triclosan treatment on persister levels.

For these experiments, we focused on ciprofloxacin, the broad-spectrum antibiotic used to treat E. coli-related UTIs. Consistent with the results of the endpoint assay (Fig. 1), triclosan substantially protected E. coli from ciprofloxacin-induced cell death throughout the duration of the time course (Fig. 2a and b). Protection was particularly pronounced at the 2-h time point, where the slope of the kill curve for pretreated cells diverged substantially from that of untreated cells (Fig. 2A). A reduced kill rate suggests that the pretreated population contains a larger proportion of persister cells (22).

In agreement with previous work (16), persister population size was proportional to the concentration of ciprofloxacin. 10% of triclosan-treated MG1655 cells cultured in 100 ng/ml ciprofloxacin remained viable after 2 h (Fig. 2A), while only 0.1% cultured in the more clinically relevant 1,000 ng/ml ciprofloxacin were viable at the same time point (Fig. 2B). For perspective, 0.1% is 1,000-fold higher than the expected frequency of persisters in an untreated population (10). After 20 h, 90,000 CFU/ml were viable in cultures treated with both triclosan and 100 ng/ml ciprofloxacin (Fig. 2A) compared to 20 cells/ml in cultures treated with ciprofloxacin alone. At 1,000 ng/ml ciprofloxacin, cultures treated with both triclosan and ciprofloxacin contained 30 viable cells per ml (Fig. 2B). In contrast, we were unable to detect viable cells (<10 cells/ml) in cultures treated with 1,000 ng/ml ciprofloxacin alone. We observed an increase in the abundance of persisters at drug concentrations above 1,000 ng/ml (see Fig. S1 in the supplemental material) (16). Although this finding initially appeared counterintuitive, it is consistent with previous reports suggesting that prophage induction in response to DNA damage is responsible for cell death at lower concentrations of ciprofloxacin, while higher concentrations kill bacteria before prophage are induced (16).

### Triclosan-mediated tolerance requires ppGpp.

Based on the well-established connection between defects in fatty acid synthesis and accumulation of ppGpp, we speculated that triclosan mediated tolerance was dependent on synthesis of the alarmone (15). To test this idea, we compared the relative viability of wild-type E. coli to mutants unable to synthesize the alarmone (ppGpp0; *spoT*::*cat* Δ*relA*) 2 h after antibiotic challenge in the presence or absence of triclosan.

**FIG 3:**
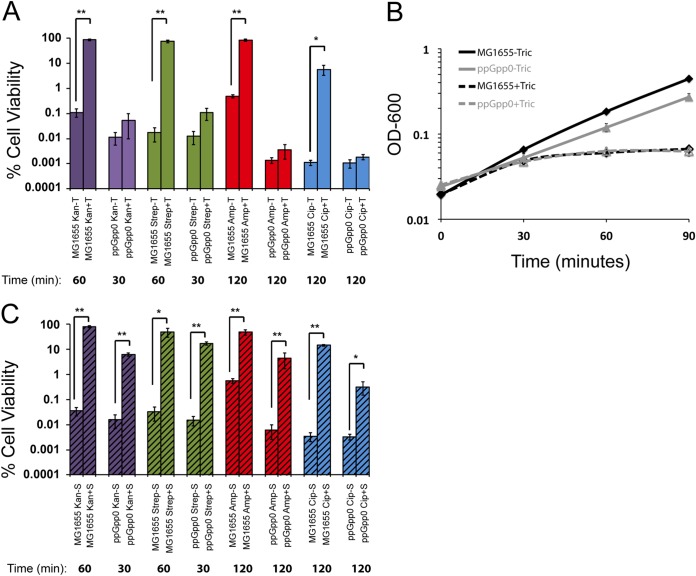
ppGpp is needed for triclosan induced tolerance. (A) Cell viability of MG1655 and ppGpp0 E. coli with (+T) or without (–T) pretreatment with triclosan after challenge with antibiotic. (B) Growth curves of MG1655 (black curve) or ppGpp^0^ (gray curve) in LB with (dashed lines) or without (solid lines) triclosan. (C) Cell viability of MG1655 and ppGpp0 E. coli with (+T) or without (–T) pretreatment with spectinomycin after challenge with antibiotic. Values are means of three independent biological replicates, with error bars representing the SEM. Asterisks represent significant differences between triclosan-treated and nontreated samples calculated using a Student two-tailed *t* test (*, *P* < 0.05; **, *P* < 0.001).

Although 200 ng/ml triclosan was sufficient to inhibit the growth of both wild-type and ppGpp0 cells, it was unable to substantially protect ppGpp0 cells from any of the four bactericidal antibiotics we tested. These include ampicillin and ciprofloxacin, as well as the translation inhibitors, kanamycin and streptomycin (Fig. 3A and B). The ppGpp0 cells were more sensitive to kanamycin and streptomycin, showing no viable cells after a 60-min treatment; thus, measurements were performed at 30 min. Importantly, triclosan alone was not bactericidal to either wild-type or ppGpp0 cells (Fig. S2).

In contrast to triclosan, pretreatment with another bacteriostatic compound, spectinomycin, increased tolerance to kanamycin, streptomycin, ampicillin, and ciprofloxacin in both wild-type and ppGpp0 mutant cells (Fig. 3C). A translation inhibitor, spectinomycin, does not impact ppGpp levels in E. coli (21). Although spectinomycin was still protective in the ppGpp0 cells, levels of protection were slightly decreased compared to wild-type cells.

### Triclosan drives tolerance to ciprofloxacin in a murine model.

Due to its widespread use and inherent stability, triclosan is present in both human populations and the environment at concentrations high enough to inhibit bacterial growth (8). Thus, a key question is whether the tolerance we observed *in vitro* is relevant *in vivo*. To determine the physiological relevance of triclosan-mediated tolerance, we employed a mouse model of E. coli UTI. UTIs are one of the most prevalent bacterial infections, impacting approximately 150 million people annually (23). Uropathogenic E. coli is the main causative agent of both uncomplicated and complicated UTI (24). Pretreatment with triclosan rendered the well-characterized E. coli cystitis isolate UTI89 ∼10-fold more tolerant to 1,000 ng/ml ciprofloxacin than untreated cells at 2 h, a level equivalent to the tolerance we observed for E. coli MG1655 at the same time point (Fig. 1 and Fig. S3).

For *in vivo* experiments, we provided 6-week-old female wild-type C3H/HeN mice with drinking water containing 1,000 ng/ml triclosan for 21 days. Control mice were given plain water for the same duration. At 21 days, experimental and control mice were transurethrally infected with ∼5 × 10^7^ CFU of E. coli UTI89. At 24 h postinfection, a subset of the mice was treated with intraperitoneal ciprofloxacin (25 mg/kg). At 48 h postinfection, all mice were sacrificed, and bacterial colonization was assessed in the urine and bladder.

**FIG 4:**
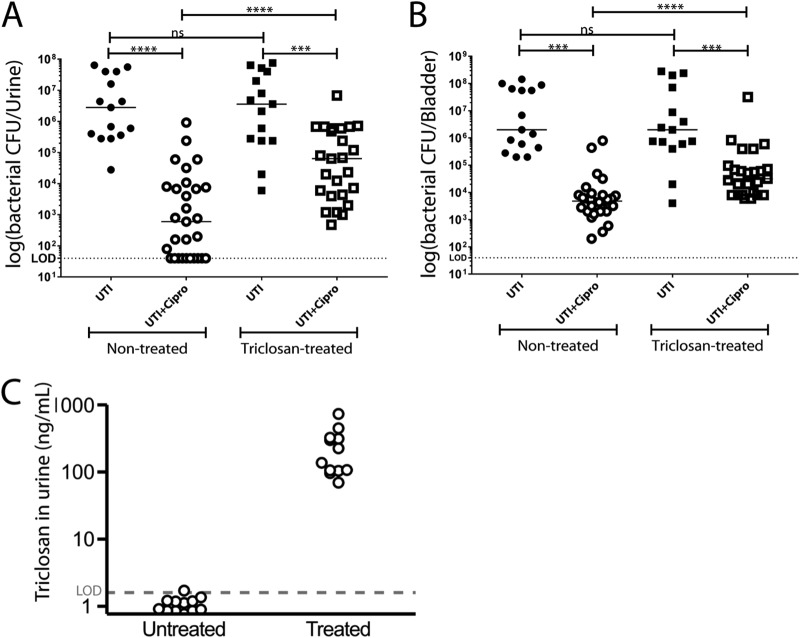
Triclosan reduces ciprofloxacin efficacy up to 100-fold in a mouse UTI model. For each round of the experiment, 15 mice were given water-containing triclosan (100 μg/ml) for 21 days, and 15 control mice received plain water. At 21 days, all mice were infected with E. coli UTI89 (∼5 × 10^7^ CFU). At 24 h postinfection, 10 mice from each group were given 25 mg/kg of ciprofloxacin intraperitoneally. At 48 h postinfection, urine samples (A) were obtained, and then the mice were sacrificed and bladders (B) were harvested. To compare the mice groups, Mann-Whitney U test was used, a *P* value of <0.05 was considered statistically significant (*, *P* < 0.05; **, *P* < 0.005; ***, *P* < 0.0005; ns, values were not statistically different). The horizontal bar represents the median value. The horizontal broken line represents the limit of detection of viable bacteria. The data are from three independent experiments. (C) Free triclosan levels measured by LC-MS/MS of triclosan in untreated and treated mouse urine. (A total of 45 mice were untreated, and 45 mice were treated; these mice were divided into three independent experiments. Urine samples from three to four mice were pooled for triclosan analysis.) The horizontal broken line represents the limit of quantification of triclosan.

After ciprofloxacin treatment, bacterial titers were >100-fold higher in the urine (*P* < 0.0001) and >10-fold higher (*P* < 0.0001) in the bladders of triclosan-treated mice versus control animals (Fig. 4A and B), consistent with triclosan-induced tolerance occurring *in vivo*. The bacterial load at 24 h postinfection was nearly equivalent in triclosan-treated and control mice, indicating that triclosan did not significantly impair UTI89 viability (Fig. 4A and B). Treated mice had triclosan levels between 70 and 750 ng/ml in their urine, a finding comparable to the MIC for E. coli (200 ng/ml) and similar to reported triclosan levels in human urine (2.4 to 3,790 ng/ml) (8) (Fig. 4C). We also detected two putative metabolized forms of triclosan, one with a mass consistent with the previously reported sulfonated triclosan (25) and the other 96 Da larger (Fig. S4). Whether or not the modified forms of the drug are active against bacteria is unclear. Control mice had no observable triclosan (below 1.6 ng/ml limit of quantification).

## DISCUSSION

Our data indicate that environmentally relevant concentrations of triclosan reduce antibiotic efficacy as much a 100-fold *in vivo* (Fig. 4) and highlight an unexpected and potentially important role for triclosan as a contributor to antibiotic tolerance and bacterial persistence in both community and health care settings. Triclosan-mediated tolerance in E. coli is dependent on ppGpp synthesis, most likely in response to inhibition of fatty acid synthesis (Fig. 3A) (15). This finding is consistent with prior work implicating ppGpp in antibiotic tolerance and persister development (16).

In contrast to previous studies of ppGpp-induced persistence that relied on either carbon starvation or the addition of serine hydroxamate to induce accumulation of high concentrations of ppGpp (100× above baseline) (26, 27), defects in fatty acid synthesis have at best a modest impact on ppGpp levels (∼5× over baseline) (15). This suggests that even relatively low levels of ppGpp are sufficient to protect cells from a panel of antimicrobials. Specifically how modest increases in ppGpp might confer tolerance to different antibiotics thus remains an open question.

Antibiotic persisters are generally divided into two categories: the trigger-induced type I and the stochastic type II. Research on the type I persisters has generally focused on entry into stationary phase as the triggering factor (10). Our data support triclosan as a separate trigger, putting triclosan-induced persistence under the type I umbrella. While previous work on both type I and II persisters identified links between growth rate and persistence, it is unclear whether reductions in growth were causative or merely correlative (10). We found that triclosan inhibits cell growth independent of ppGpp production (Fig. 3B), while triclosan-mediated changes in antibiotic tolerance and persistence require a functional ppGpp response (Fig. 3A). Together, these data support a model in which persistence and tolerance are complex phenomena, likely dependent on multiple growth-dependent and growth-independent factors.

We favor the idea that triclosan induces tolerance in wild-type cells primarily via its indirect, positive impact on intracellular ppGpp levels and the concomitant downregulation of global biosynthesis. Triclosan inhibits fatty acid synthesis, reducing activity of the SpoT hydrolase and increasing intracellular ppGpp concentration (20). ppGpp in turn inhibits flux through multiple biosynthetic pathways, increasing tolerance to cognate antimicrobials. For example, in this model ppGpp-dependent reductions in DNA replication and protein synthesis reduce sensitivity to ciprofloxacin and kanamycin, compounds that target DNA gyrase and the ribosome, respectively (28–30). While triclosan inhibits fatty acid synthesis and restricts growth in wild-type and ppGpp0 mutants alike, DNA replication, translation, and cell wall synthesis likely remain at least partially intact in the absence of ppGpp, rendering the mutants sensitive to antibiotics targeting those pathways (31). In contrast, spectinomycin inhibits protein synthesis directly, resulting in a general, ppGpp-independent downregulation of biosynthesis and an increase in tolerance to drugs targeting downregulated processes, even in the ppGpp0 cells (Fig. 3C).

*In vivo*, triclosan may also contribute to tolerance by promoting attachment and biofilm formation. Bacteria in biofilms typically exhibit enhanced antibiotic tolerance, in part due to their high cell density and reduced metabolic rate (32). Increases in ppGpp concentration are associated with enhanced biofilm formation in E. coli (33), and triclosan has been shown to increase surface attachment and biofilm formation in S. aureus (34).

An open question is the impact of triclosan on tolerance in other bacterial systems. Triclosan increases tolerance in S. aureus *in vitro* (Fig. 2C), although the dependence on ppGpp remains an open question. While S. aureus can cause UTIs, it predominantly infects the skin, where residual triclosan concentrations likely vary substantially and quantification is significantly more challenging than urine (35). It is, however, worth noting that studies of the nasal cavity have identified levels of triclosan high enough to potentially induce tolerance (34).

Prescribing a bacteriostatic compound prior or along with delivery of a bactericidal one is generally recognized as poor practice (36) due to the potential of the former interfering with the activity of the other. At the same time, bacteriostatic mechanisms of action, and thus the mechanisms by which these drugs drive tolerance are likely to differ widely. While the translation inhibitor spectinomycin provides protection against bactericidal compounds, it does not induce accumulation of ppGpp (21), a fact supported by our finding that spectinomycin induces tolerance to bactericidal compounds in both wild-type and ppGpp0 cells (Fig. 3c). At the same time, ppGpp-dependent induction of antibiotic tolerance is likely to be a feature triclosan shares with a related compound, triclocarban, which similarly inhibits an early step in fatty acid synthesis and is also a common additive in consumer products. Triclosan also stands out from other bacteriostatic compounds by virtue of its widespread use and sheer abundance in the environment. ∼1 kg of triclosan is produced for every 3 kg of other antimicrobials and estimates indicate that ∼100 metric tons are being deposited annually in the environment through wastewater treatment in the United States alone (37).

Although triclosan has low toxicity (50% lethal dose = 4,350 mg/kg orally) (38), accumulating data link long-term exposure with antibiotic resistance (39), and there are reports that triclosan may also function as an endocrine disrupter (40, 41). Our analysis of the impact of triclosan on antibiotic efficacy in a mouse UTI model (Fig. 4) highlights yet another deleterious “side effect” of this ubiquitous antimicrobial. UTIs alone impact 150 million people worldwide (23) at a cost of $3.5 billion per year in the United States alone (42). Complications associated with UTIs include pyelonephritis with sepsis, renal damage, preterm birth, Clostridium difficile colitis, sepsis, and death, particularly in the very old and the very young (24). Coupled with the well-established connection between antibiotic tolerance and recurrent/chronic infections (12, 13), our findings reinforce the need for substantial caution—as well as consideration of unintended consequences—in evaluating the costs and benefits of antimicrobial additives in consumer products.

## MATERIALS AND METHODS

### Materials and strains.

Triclosan, ampicillin, kanamycin, streptomycin, ciprofloxacin, and vancomycin were purchased from Sigma-Aldrich. Stock solutions were made in water for ampicillin (100 mg/ml), kanamycin (50 mg/ml), streptomycin (100 mg/ml), and ciprofloxacin (10 mg/ml). Triclosan was dissolved in ethanol (10 mg/ml), and vancomycin was dissolved in dimethyl sulfoxide (100 mg/ml). E. coli MG1655 and S. aureus FPR3575 were both lab strains, and E. coli UTI89 was isolated from a patient with a UTI (43). E. coli was grown in Luria-Bertani broth (LB), and S. aureus was grown in tryptic soy broth (TSB). Growth temperature was 37°C for all experiments.

### Determination of MIC (MIC).

To determine the MIC for the panel of antibiotics utilized in this study, E. coli and S. aureus were grown to optical density at 600 nm (OD_600_) of 0.1 in LB or TSB, respectively. Cells were then back-diluted 1,000-fold and transferred to a 96-well plate containing 2-fold dilutions of respective antibiotics and cultured at 37° for 16 additional hours with vigorous shaking in a BioTek Eon plate reader. MIC was calculated as the lowest antibiotic concentration preventing development of detectable turbidity at OD_600_.

### Assays for antibiotic tolerance and persistence.

To assay tolerance and persistence, E. coli and S. aureus were grown to an OD_600_ of 0.2 in LB or TSB, respectively. Cells were then back-diluted to an OD_600_ of 0.1 in medium containing triclosan at the indicated concentrations and cultured for an additional 30 min before being challenged with bactericidal antibiotics. For dot plating, 10 μl of a 10-fold dilution series was plated on antibiotic-free LB-agar or TSB-agar as appropriate. For determination of the CFU, 100 μl of a 10-fold dilution series was spread on antibiotic free LB-agar or TSB-agar plates. Cells were incubated for ∼12 h at 37°C prior to quantification. CFU were normalized to CFU at the initial time point to correct for the ∼2-fold increase in cell number in untreated cultures during the 30 min pretreatment period. The relative persistence is defined as the CFU of the triclosan-treated sample divided by the CFU of the nontreated sample.

### UTI mouse work.

Six-week-old female wild-type C3H/HeN mice were obtained from Envigo. Mice were treated with or without 100 μg/ml triclosan (100 ppm) in the drinking water for 21 days. At 21 days, the mice were anesthetized by inhalation of 4% isoflurane, and mouse bladders were transurethrally infected with approximately 5 × 10^7^ CFU of E. coli UTI89 in 50 μl of PBS (44). Briefly, a single UTI89 colony was inoculated in 20 ml of LB and incubated at 37°C under static conditions for 24 h. Bacteria were then diluted (1:1,000) into fresh LB and incubated at 37°C under static conditions for 18 to 24 h. Bacteria were subsequently washed three times with phosphate-buffered saline (PBS) and then concentrated to approximately 5 × 10^7^ CFU per 50 μl. At 24 h postinfection, mice received 25 mg/kg of ciprofloxacin intraperitoneal. At 48 h postinfection, mice were euthanized, bladders were harvested, and urine samples were collected. Bladders were homogenized in PBS, and the bacterial load present in bladders and urine samples was determined by plating serial dilutions on LB-agar supplemented with antibiotics when appropriate. Statistical analyses were performed using a Mann-Whitney U test with GraphPad Prism software (v6.0 for Mac). All animal studies were performed in accordance with the guidelines of the Committee for Animal Studies at Washington University School of Medicine.

### Measurement of triclosan and metabolites in mouse urine.

Since triclosan has been observed to adsorb to plastic surfaces, sample handling was performed in glass vessels whenever possible (25). A stock solution of 1 mg/ml triclosan (Sigma) was prepared in methanol and a 100 μg/ml ^13^C_12_-triclosan (99%) internal standard in methyl *tert*-butyl ether was purchased from Cambridge Isotope Laboratories (Andover, MA). A dilution series of 1,000, 200, 40, 8, 1.6, and 0.32 ng/ml triclosan was prepared in pooled, untreated mouse urine and spiked with 100 ng/ml ^13^C_12_-triclosan internal standard. Samples were diluted 1:1 in methanol, spun down at 20,000 × *g* for 10 min, and filtered through 0.45-μm-pore-size, 13-mm-diameter polyvinylidene difluoride syringe filters (Millipore). Finally, cleaned samples were diluted 1:1 in high-pressure liquid chromatography-grade water (Sigma).

Using a Shimadzu UFLC (Kyoto, Japan), 10 μl of each sample was injected onto a fused core phenyl-hexyl column (100 mm × 2 mm × 2.7 μm) with a 0.4 ml/min flow rate (Ascentis Express; Supelco). Triclosan was eluted from the column as follows: solvent A (0.1% formic acid) and solvent B (90% acetonitrile with 0.1% formic acid) were held constant at 80 and 20%, respectively, for 0.1 min. Solvent B was increased to 98% by 5 min, held at 98% for 1 min, and then reduced again to 20% in 0.1 min. The column was equilibrated in 20% solvent B for 3 min between runs.

Triclosan was detected using an AB Sciex API 4000 QTrap mass spectrometer (AB Sciex, Foster City, CA) running in negative ion electrospray ionization mode (ESI) using a Turbo V ESI ion source. Triclosan was detected using the instrument settings listed in Table S1 in the supplemental material. A precursor ion scan was performed for the 35 *m/z* product ion to determine the mass spectrum of triclosan, ^13^C_12_-triclosan, and any potential metabolites (Fig. S4a). Because triclosan contains three chlorine atoms, its mass spectrum includes prominent isotope peaks (M+2 and M+4) corresponding to the natural abundance of ^37^Cl (Fig. S4b). To improve sensitivity, product ions from the two most abundant isotopologues were detected and added together prior to peak integration. Peaks for triclosan and internal standard were integrated with Analyst software (AB Sciex) and normalized. Normalized peak areas varied linearly with triclosan concentration above 1.6 ng/ml.

Pooled urine samples from three to four mice were spiked with 100 ng/ml internal standard and cleaned as described above. Samples were analyzed by liquid chromatography-tandem mass spectrometry (LC-MS/MS), and triclosan was quantified using a standard curve (Fig. S4e).

### Statistical analysis.

Values for the *in vitro* data are expressed as means ± the standard errors of the mean (SEM) from *n* = 3 replicates. *In vitro* data were analyzed using a two-tailed Student *t* test with statistical significance determined when the *P* value was <0.05. For the mouse data, a Mann-Whitney U test was used to test for statistical significance. Values represent means ± the SEM derived from at least three independent experiments (*, *P* < 0.05; **, *P* < 0.005; ***, *P* < 0.0005; ****, *P* < 0.00005; ns, difference not significant).

## Supplementary Material

Supplemental file 1
