# Supplementary material for: The Widely Used Antimicrobial Triclosan Induces High Levels of Antibiotic Tolerance In Vitro and Reduces Antibiotic Efficacy up to 100-Fold In Vivo
Source: Antimicrob Agents Chemother. 2019 Apr 25;63(5):e02312-18. doi: 10.1128/AAC.02312-18 (PMC6496070; doi:10.1128/AAC.02312-18)
Supplement: Supplemental file 1 [file AAC.02312-18-s0001.pdf]

Supplementary Data

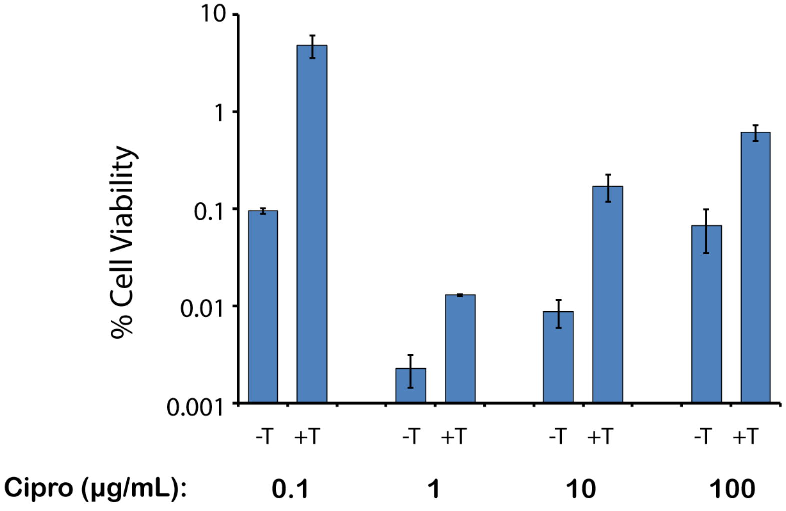

Supplementary Data Figure 1. Triclosan protects at high concentrations of ciprofloxacin. MG1655 cells were grown to OD600 = 0.1 before triclosan was added for a final concentration of 200 ng/mL for 30 minutes. Ciprofloxacin was added at the labeled concentration. Ciprofloxacin was washed off, and cell viability was determined. Values are shown as averages of three replicates with error bars showing the standard error of the mean.

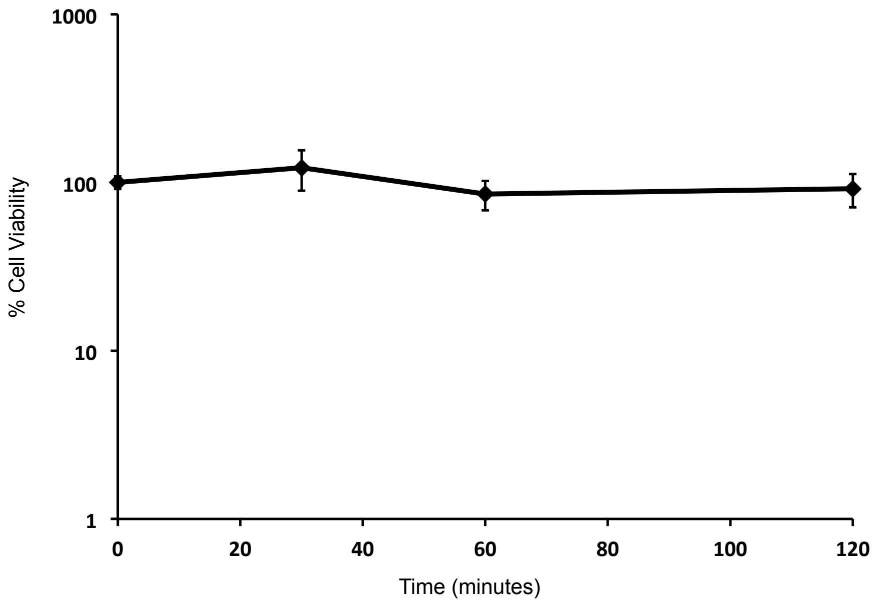

Supplementary Data Figure 2. Triclosan is not bactericidal to the ppGpp0 cells. ppGpp0 cells were grown to OD600 = 0.2 before triclosan was added for a final concentration of 200 ng/mL. Cells were plated at each time pointed and colony-forming units were quantified. Each point represents the average of three biological replicates with the error bars representing the standard error of the mean.

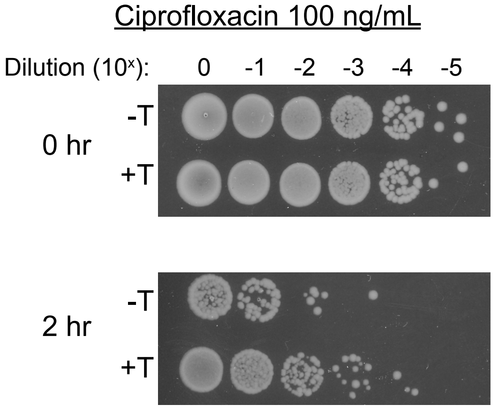

Supplementary Data Figure 3. Triclosan induces ciprofloxacin tolerance in the uropathogentic *E. coli* UTI89. UTI89 cells were grown up to OD600 = 0.2, split and cultured for an additional 30 minutes with (+T) or without 200ng/ml triclosan (-T). Ciprofloxacin was added to obtain a final concentration of 100 ng/mL. Cells were dot-plated at the 0- and 2-hour time points. The plating efficiency was repeated three independent times with a representative image shown.

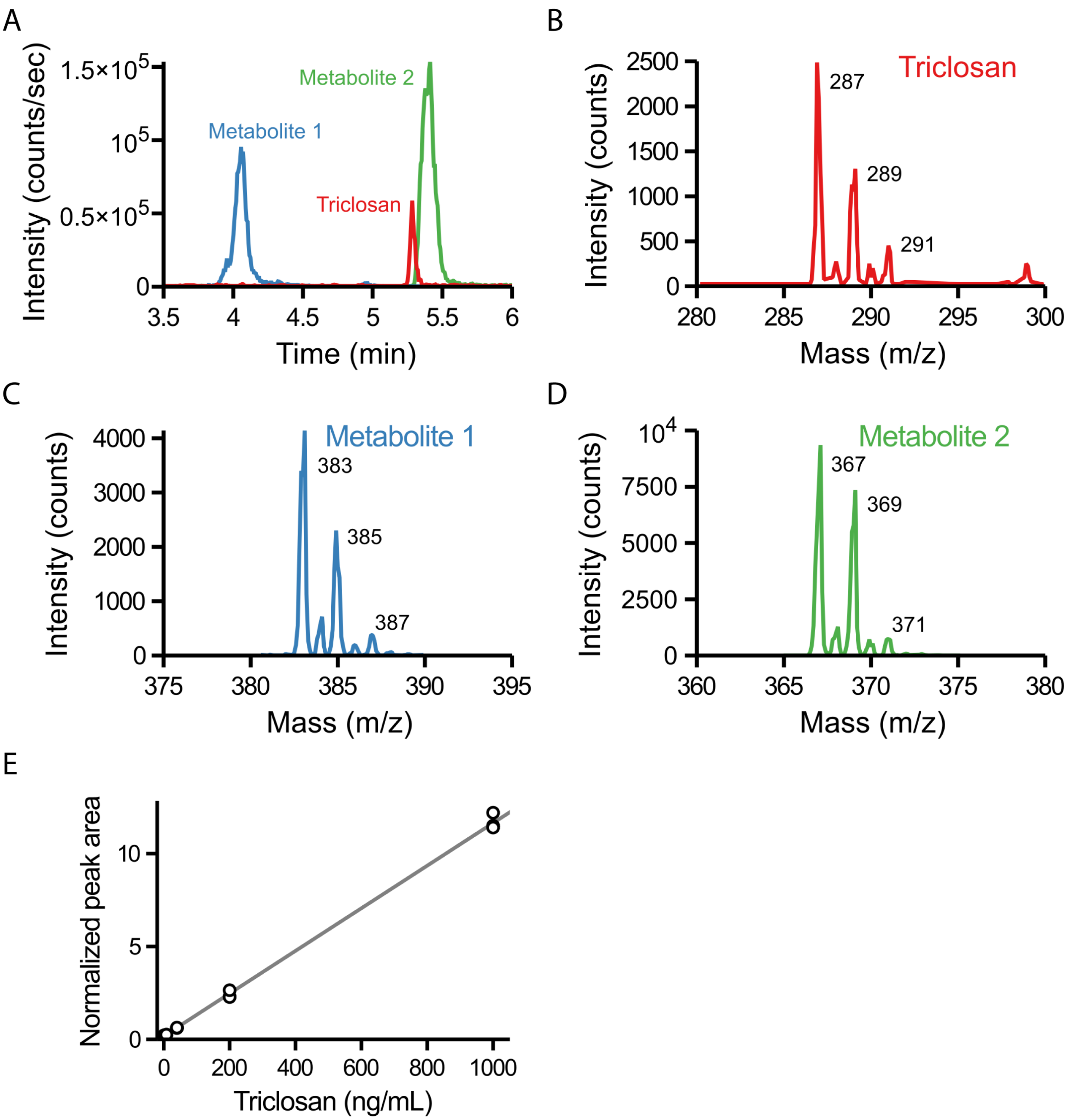

Supplementary Data Figure 4. Measurement of free and metabolized triclosan in triclosan-treated mouse urine. Chromatogram of a representative LC-MS/MS experiment showing the elution profile of metabolite 1 (blue), triclosan (red) and metabolite 2 (green) (A). Mass spectra

of triclosan, metabolite 1, and metabolite 2, respectively, measured using precursor ion scans for the chloride product ion (35 m/z) (B,C, and D). Triclosan peak area detected by LC-MS/MS varies linearly with concentration down to 1.6 ng/mL (E).

Instrument Settings		
Ion Spray Voltage	-4.5 kV	
Heater Temperature	500 °C	
Nebulizer Gas	40	
Auxiliary Gas	40	
Declustering Potential	-10 V	
Collision Energy	-40 V	
**Ions Detected (m/z)**		
Triclosan	286.8 → 35	288.7 → 35
$^{13}C_{12}$-Triclosan	298.84 → 35	300.74 → 35
Metabolite 1	382.8 → 35	384.8 → 35
Metabolite 2	366.7 → 35	368.8 → 35

Supplementary Data Table 1. MS/MS settings for triclosan detection
